# Establishing a Baseline for Human Cortical Folding Morphological Variables: A Multisite Study

**DOI:** 10.3389/fnins.2022.897226

**Published:** 2022-07-18

**Authors:** Fernanda H. P. de Moraes, Victor B. B. Mello, Fernanda Tovar-Moll, Bruno Mota

**Affiliations:** ^1^Brain Connectivity Unit, Instituto D'Or de Pesquisa e Ensino, Rio de Janeiro, Brazil; ^2^metaBIO, Instituto de Física, Universidade Federal do Rio de Janeiro, Rio de Janeiro, Brazil

**Keywords:** cortical folding, aging, Alzheimer's Disease, harmonization, baseline estimation

## Abstract

Differences in the way human cerebral cortices fold have been correlated to health, disease, development, and aging. However, to obtain a deeper understanding of the mechanisms that generate such differences, it is useful to derive one's morphometric variables from the first principles. This study explores one such set of variables that arise naturally from a model for universal self-similar cortical folding that was validated on comparative neuroanatomical data. We aim to establish a baseline for these variables across the human lifespan using a heterogeneous compilation of cross-sectional datasets as the first step to extending the model to incorporate the time evolution of brain morphology. We extracted the morphological features from structural MRI of 3,650 subjects: 3,095 healthy controls (CTL) and 555 patients with Alzheimer's Disease (AD) from 9 datasets, which were harmonized with a straightforward procedure to reduce the uncertainty due to heterogeneous acquisition and processing. The unprecedented possibility of analyzing such a large number of subjects in this framework allowed us to compare CTL and AD subjects' lifespan trajectories, testing if AD is a form of accelerated aging at the brain structural level. After validating this baseline from development to aging, we estimate the variables' uncertainties and show that Alzheimer's Disease is similar to premature aging when measuring global and local degeneration. This new methodology may allow future studies to explore the structural transition between healthy and pathological aging and may be essential to generate data for the cortical folding process simulations.

## 1. Introduction

Mapping the human brain development and aging longitudinally from a unique dataset is barely impossible due to our extensive lifespan. Suppose one wants to understand the time evolution of the brain morphology through the whole lifespan. In this case, it is mandatory to combine multiple datasets and devise methods that allow a fair comparison among them. The recent advent of large heterogeneous datasets (to ensure the legit results across several populations) and data curation innovations allowed neuroscience to explore the brain's changes on an enormous scale, from functional data to structural studies. Here, we propose a novel methodology of combining structural MRI from different acquisition sites and equipment, acknowledging their heterogeneity and providing insights about cortical folding during development and aging, with a practical application of these results in Alzheimer's Disease, the most common dementia worldwide. This effort is essential to provide experimental information about the evolution span of the cortex morphology and build a cortical folding theory.

In the past years, the study of cortical folding in humans and other mammals was extensively promoted by the application of translational science, open-access databases, and data science tools which allowed the field to grow in multiple and sometimes convergent directions (Madan and Kensinger, 2016; Llinares-Benadero and Borrell, 2019; Mota et al., 2019; Essen, 2020). Due to its biological background, cortical folding measurements have been included in human brain structure analysis, discriminating disease from healthy controls (Cao et al., 2017; Lamballais et al., 2020), describing its correlation with cognition (Núñez et al., 2020), and relation to aging (Hogstrom et al., 2013; Madan, 2021). Despite the lack of consensus defining a unique theory that explains cortical folding on every scale. Mota and Herculano-Houzel (2015) proposed a cortical folding model respected by more than 50 mammals' brain hemispheres. It predicts a power-law relationship between cortical thickness (*T*), exposed (*A*_*E*_), and total area (*A*_*T*_) (Equation 1)


(1) $$\begin{array}{r} T^{\frac{1}{2}}A_{T}=kA_{E}^{\alpha } \end{array}$$

where α is the self-similarity index, a universal constant, with a theoretical value of 1.25 and calculated as 1.305 (Mota and Herculano-Houzel, 2015) when considering a heterogeneous dataset of different species. It is an index of complexity, reflecting how much detail in a real fractal pattern changes with the scale at which it is measured. The constant *k* is dimensionless and associated with conserved viscoelastic properties of cortical matter. Following this equation, the usual geometrical variables used to describe the cortex (*T*, *A*_*T*_, and *A*_*E*_) are not independent in this model.

Wang et al. continued this study, validating the theory for different groups of humans (Wang et al., 2016) in specific regions of interest (Wang et al., 2019b), obtaining new evidence that the cortex is indeed a self-similar structure. Wang et al. (2021) showed that from this model one could derive a more natural set of nearly independent morphological variables, K, S, and I, that could be used to improve disease discrimination from regular and typical structural changes of the brain, such as aging.

In this framework, the morphology of each cortex is expressed as a point in a three-dimensional abstract space, with each coordinate component corresponding to the log-value of an independent morphometric variable (Wang et al., 2021). The use of log-values guarantees that linear combinations of the basis vectors correspond to power-law relations. Conversely, as long as any new variables are expressible as power-laws, different but equivalent sets of variables can, thus, be related to each other by a change of Cartesian coordinate bases. As a starting point, we use the log-values of the commonly morphometric variables, the total area log_10_*A*_*T*_, exposed area log_10_*A*_*E*_ and average thickness $\log_{10}T^{2}$. We then derive our new variables thusly: *K* = log_10_*k* (Equation 2) is a near-invariant quantity obtained by isolating *k* in Equation (1). S (Equation 3), also dimensionless, encapsulates the aspect of brain shape that changes more significantly across cortices, and I (Equation 4), represents brain isometric volume and carries the information about overall cortical size. Both are derived from planes perpendicular to *K*, noting that *K* remains unchanged when applying an isometric transformation on the brain (a direction in which all areas scale equally) resulting in *I*, and the perpendicular dimensionless vector *S* resultant from *K* × *I*^1^ ^2^. Those three variables combined may help distinguish pathological events similar to age effects, such as AD.


(2) $$\begin{array}{r} K=\log_{10}k=\log_{10}A_{T}-\frac{5}{4}\log_{10}A_{E}+\frac{1}{4}\log_{10}T^{2} \end{array}$$

(3) $$\begin{array}{r} S=\frac{3}{2}\log_{10}A_{T}-\frac{3}{4}\log_{10}A_{E}-\frac{9}{4}\log_{10}T^{2} \end{array}$$

(4) $$\begin{array}{r} I=\log_{10}A_{T}+\log_{10}A_{E}+\log_{10}T^{2} \end{array}$$

Baseline values (age-specific norm values) of those cortical morphological variables and their inherent uncertainties over the human lifespan were never defined due to methodological limitations of combining multicenter studies of brain structural images (Gronenschild et al., 2012; Fortin et al., 2018). However, both are crucial for applied studies. Uncertainties determine measuring limitations and allow a proper comparison between different cohorts. Baseline values allow clinical applications and, when necessary, simulations of healthy control data. Multi-center studies have become more common in the last few decades due to improved data sharing and the open science trend. However, combining MRI data from multiple experiments is not a trivial task. Part of these uncertainties is explained by random errors, a direct consequence of the natural variance within the species individuals, and the impossibility of replicating the exact same condition over multiple data acquisitions. Theoretically, these random errors are the same if one considers multiple experiments.

Confounding components could be added when gathering MRI images with differences in acquisition parameters, acquisition equipment, and versions of the post-processing software (Dickerson et al., 2008; Gronenschild et al., 2012). Together, they add uncertainty to the data due to a systematic effect, ultimately reflecting a systematic difference in morphological variables calculated from different databases. In practice, the natural random fluctuations are convoluted with the uncertainty due to a systematic effect, enhancing the data spread.

Recently, a vast literature on primary morphological variables from MR images (Schnack et al., 2010; Pomponio et al., 2020; Bethlehem et al., 2022), and more complex ones (Fortin et al., 2018), explored the limitations of compiling images acquired with different scanners and protocols and its implication in statistical analysis results. The suggested solution for these limitations is a procedure called data harmonization, in which there is an “explicit removal of site-related effects in multi-site data” (Pomponio et al., 2020).

It is essential to add that one cannot, in general, distinguish the natural variance in healthy human anatomy (σ_Natural_) and data acquisition random errors (σ_Random_). Also, it is not possible to distinguish between the uncertainty due to a systematic effect of the acquisition and the processing. In other words, any measurement setup can include an additional value at its final measure that is repeated for all measurements. Here, the acquisition effects are due to differences in MR structural images acquisition protocols and their parameters, while processing errors are due to differences in the software version, pipeline parameters, and computer used for processing (Gronenschild et al., 2012). In Fortin et al. (2018) acquisition and processing effects are described as *scanner and site effects*^3^. However, in the case of the methodology of this manuscript, it is impossible to quantify the contribution of this systematic shift, from acquisition and processing, since we did not compare processing or acquisition parameters by controlling the other error sources. For this, one would need a diligent procedure to track all differences between acquisition and processing as well as multiple image of the same individual for each sample. This manuscript deals only with the first case, approximating the random errors due to acquisition and processing for all samples using three subsequent structural images of the subjects available only for one particular sample (refer to Section Supplementary Material Section 2). The total uncertainty associated with the variables (K, S, and I) and their components is then summarized in Equation (5), the error propagation formula with no correlation since we are treating errors from uncorrelated sources by definition.


(5) $$\begin{array}{r} \sigma_{X}^{2}=\sigma_{Natural}^{2}+\sigma_{Random}^{2}+\sigma_{Sample}^{2} \end{array}$$

At which σ_*X*_, is the total uncertainty for K, S, and I, σ_*Natural*_ represents the so-called here Natural Fluctuation, σ_*Random*_, the random variance that can be evaluated from the repeated measures with the same subject at each Sample acquisition and processing steps, and σ_*Sample*_, for the uncertainty due to a systematic effect on each sample.

In this manuscript, we developed a simplistic harmonization method that allows a multi-site analysis, determining K, S, and I baseline values, their uncertainties, and the ratio of changing rates through the years for cross-sectional images of healthy controls. Although this work focused is these novel morphological variables, we also provided the baseline of usual morphological variables such as cortical thickness (*T*) and Gyrification Index (*GI*). A robust estimation framework of a baseline time function is important to provide future clinical studies with enough information to compare brain structure trajectories in case of a pathological investigation. We then, as a clinical application and extension of de Moraes et al. (2022) and Wang et al. (2019b), verify if Alzheimer's Disease (AD) is similar to a premature accelerated aging brain in terms of the independent morphological variables, K, S, and I, by comparing their rates. Finally, we have determined the time evolution of the self-similarity dimension α proposed in the Mota and Herculano-Houzel (2015) model, interpreting all results within the framework of this theory.

## 2. Materials and Methods

Participants and data included in this analysis were either acquired by the Instituto D'Or de Pesquisa e Ensino (IDOR), Rio de Janeiro, Brazil; used at de Moraes et al. (2022) (approved by the Hospital Copa D'Or Research Ethics Committee under protocol number CAAE 47163715.0.0000.5249); shread datasets included at Wang et al. (2016, 2019b), as the Alzheimer's Disease Neuroimaging Initiative (ADNI) database (adni.loni.usc.edu), the Human Connectome Project (HCP), Information eXtraction from Images (IXI), Nathan Kline Institute (NKI) and Open Access Series of Imaging Studies (OASIS) Wang et al. (2016, 2019b); or from open-access databases, as Ultra-high field adult lifespan (AHEAD) (Alkemade et al., 2020) and (Snoek et al., 2021). The total number of subjects is 3,095 healthy controls from 4 to 96 years old and 555 Alzheimer's Disease subjects from 56 to 92 years old. Datasets' demographics are summarized in Table 1. AHEAD and HCPr900 only include age range instead of the actual age due to local Ethics Committee rules. To overcome the lack of an exact number, we assumed the interval's mean age as a reference.

**Table 1:** Summary for each dataset.

**Datasets**	**Diagnostic**	***N* (F)**	**Age (age range) [years]**
ADNI Jack et al., 2010a	CTL	868 (445)	75 ± 6.5 (56; 96)
	AD	542 (241)	75 ± 8 (56; 92)
AHEAD Alkemade et al., 2020	CTL	100 (56)	42 ± 19 (24; 76)
AOMIC ID1000^*^ Snoek et al., 2021	CTL	50 (27)	23 ± 1.7 (20; 26)
AOMIC PIOP01 Snoek et al., 2021	CTL	208 (120)	22 ± 1.8 (18; 26)
AOMIC PIOP02 Snoek et al., 2021	CTL	224 (128)	22 ± 1.8 (18; 26)
HCP900r Glasser et al., 2013	CTL	881 (494)	29 ± 3.6 (22; +36)
IDOR	CTL	77 (53)	66 ± 8.4 (43; 80)
	AD	13 (8)	77 ± 6.1 (63; 86)
IXI-Guy's	CTL	314 (175)	51 ± 16 (20; 86)
IXI-HH	CTL	181 (94)	47 ± 17 (20; 82)
IXI-IOP	CTL	68 (44)	42 ± 17 (20; 86)
NKI Nooner et al., 2012	CTL	168 (68)	34 ± 19 (4; 85)
OASIS Marcus et al., 2010	CTL	312 (196)	45 ± 24 (18; 94)
TOTAL	CTL	3,095 (1,762)	46 ± 23 (4; 96)
	AD	555 (249)	75 ± 8 (56; 92)

* *Used only for repeated measures analysis*.

*Mean value ± SD. Diagnostic: CTL, Healthy control; AD, Alzheimer's Disease. Details in Supplementary Material*.

To investigate the uncertainty due to a systematic effect during image acquisition and variances in repeated measures performed in the same conditions, we included the first 50 subjects from the AOMIC ID1000 (Snoek et al., 2021), described in Table 1.

The structural images AHEAD, AOMIC ID1000, PIOP1, and PIOP2 were processed in FreeSurfer v6.0.0 (Fischl, 2012) without manual intervention at the surfaces (McCarthy et al., 2015). The original project from IDOR images is longitudinal. Those images in particular were processed with the longitudinal pipeline. The FreeSurfer localGI pipeline generates the external surface and calculates each vertex's local Gyrification Index (localGI) (Schaer et al., 2008). Values of Cortical Thickness, Total Area, Exposed Area, and Local Gyrification Index were extracted with Cortical Folding Analysis Tool (Wang et al., 2019a). We defined as ROI the whole hemisphere, frontal, temporal, occipital, and lateral lobes (based on FreeSurfer definition of lobes). The lobes' area measurements were corrected by their integrated Gaussian Curvature, removing the partition size effect and directly comparing lobes and hemisphere cortical folding, as described in Wang et al. (2019b).

Datasets' demographics, acquisition, and processing information are summarized in Supplementary Material.

### 2.1. Statistical Data Analysis

Multiple comparisons of means were made with ANOVA, correlations between cortical folding independent variables, and age estimated with Pearson r and *post-hoc* evaluations with Tukey multiple comparisons of means, including comparisons within Diagnostic groups. The statistical significance threshold was set at α = 0.05, and multiple corrections (Bonferroni) were applied when needed. The Healthy Control subjects are used as a reference in the necessary normalization and harmonization procedures. A standard linear regression was used to obtain the self-similarity index α.

All analyses were done considering the whole cortical hemisphere and the lobes (refer to Supplementary Material), as most diseases imply local or non-uniform global structural damage. Linear Mixed Models (LMM), standard linear regression, and statistics derived from their results were analyzed with RStudio (v1.4.1717 and R v4.1.1). The Bayesian model comparison was developed in Python3 with the package PyMC3. Harmonization and *deaging* codes are available at https://zenodo.org/record/5879895 (de Moraes et al., 2021).

### 2.2. Harmonization

The data harmonization procedure is based on the time evolution of the basic morphological variables *Y* = *T*, *A*_*T*_, or *A*_*E*_, modeled as an exponential decay with the age *t*_age_. Using Linear Mixed Models (LMM) in the log-linear scale, we are able to perform a joint linear fit for each ROI, maintaining the same angular coefficient *a* among all samples but different linear coefficient *b*_*j*, ROI_ according to Equation (6). We then subtracted the sample shift, given by the linear coefficients *b*_*j*, ROI_), directly obtained from the Sample Random Effects (*log*_10_(*Variable*) ~*Age*×*ROI*+(1|*Sample*:*ROI*)) in Healthy Controls. From this procedure, we directly calculate the variables K, S, and I and determine for all morphological variables of interest (also *T*, *A*_*T*_, *A*_*E*_, GI = *A*_*t*_/*A*_*e*_) their baseline and uncertainties. This harmonization procedure was validated as the most probable description of the data using a Bayesian approach. This simple harmonization procedure is equivalent to the approach presented in Bethlehem et al. (2022) for the special case of a purely linear function.


(6) $$\begin{array}{r} \begin{array}{c} {\text{log}}_{10}Y_{j,\text{ROI}}=at_{\text{age}}+b_{j,\text{ROI}}\text{, for each ROI and each sample j} \end{array} \end{array}$$

#### 2.2.1. Bayesian Model Selection

In order to show that the harmonization procedure used was the most suitable, one can compare it with other models to describe the data. For example, the angular coefficient could also be a random effect for the LMM, varying across the samples. Using Bayesian model selection (Gregory, 2005), one can show that the LMM used in this study is the most probable description of the data.

Considering our dataset comprised by *J* samples where *D*_(*j*)_ = {*d*_1(*j*)_, *d*_2(*j*)_, ⋯ , *d*_*N*_*j*_(*j*)_, } denotes the data of a morphological variable in the *j*-th sample. The degree of belief in a model *M* that describes a normally distributed data for each age is given by the posterior probability:


(7) $$\begin{array}{l} P(M|D_{\left(j\right)})=\int_{\Delta A_{(j)\beta }}dA_{(j)\beta }P(A_{(j)\beta }|I)\prod_{i=1}^{N_{\left(j\right)}}\frac{1}{\sigma_{\left(j\right)}\sqrt{2\pi }}\\ \;\exp{\left[y_{i(j)}-\mu \left(x_{i(j)},A_{(j)\beta }\right)\right]}^{2}/2\sigma_{\left(j\right)}^{2} \end{array}$$

(8) $$\begin{array}{r} P\left(M|D\right)=\overset{J}{\underset{j=1}{\prod }}\int_{\Delta A_{\left(j\right)\beta }}dA_{\left(j\right)\beta }P\left(A_{\left(j\right)\beta }|I\right)\overset{N_{\left(j\right)}}{\underset{i=1}{\prod }}\frac{1}{\sigma_{\left(j\right)}\sqrt{2\pi }}\\ \;\exp{\left[y_{i\left(j\right)}-\mu \left(x_{i\left(j\right)},A_{\left(j\right)\beta }\right)\right]}^{2}/2\sigma_{\left(j\right)}^{2} \end{array}$$

The term μ(*x*_*i*(*j*)_, *A*_(*j*)β_) is given by the model *M* proposed to describe the data, with the set {*A*_(*j*)β_} being the free parameters of the model defined within a range Δ*A*_(*j*)β_. A prior probability *P*(*A*_(*j*)β_|*I*) is assigned for all free parameters reflecting the prior knowledge about their values. The basic model of this study assumes that the log of morphological variables follows μ = *ax*+*b*_(*j*)_, with the dispersion σ_(*j*)_ for each sample. Uninformative priors were used for the free parameters. This integral can be easily done numerically using Python3 with PyMC3 package or similar. The advantage of this approach is that it naturally penalizes the insertion of unnecessary new parameters avoiding over-fitting.

With multiple models *M*_*i*_, one defines the odds as $O_{ij}=\frac{p\left(M_{i}|D\right)}{p\left(M_{j}|D\right)}$. As $\underset{i}{\sum }p\left(M_{i}|D\right)=1$, the probability of the model *M*_1_ is given by:


$$\begin{array}{l} p\left(M_{1}\right)=\frac{1}{1+\sum_{i}O_{i1}} \end{array}$$

The alternative models considered by this study were: (i) μ = *ax*+*b*_(*j*)_, with the same dispersion σ_(*j*)_; (ii) μ = *a*_(*j*)_*x*+*b*_(*j*)_, with the dispersion σ_(*j*)_ for each sample; (iii) μ = *a*_(*j*)_*x*+*b*_(*j*)_, with the same dispersion σ_(*j*)_ for each sample; (iv) we also tested the simplest non-linear trend $\mu =cx^{2}+ax+b_{\left(j\right)}$, with the dispersion σ_(*j*)_ for each sample; we have found that the LMM used in the study is the most probable. The code implementing this approach is given by the authors.

It is important to note that all parameters calculated using the LMM approach can be obtained in this framework by a slight modification of Equation (8). Instead of marginalizing all the parameters, by leaving one out, we obtain *P*(*A*_*j*1_|*D*) which gives directly the most probable value for the parameter *A*_*j*1_ and its credible region.

## 3. Results

### 3.1. Multisite Harmonization

**Figure 1:**
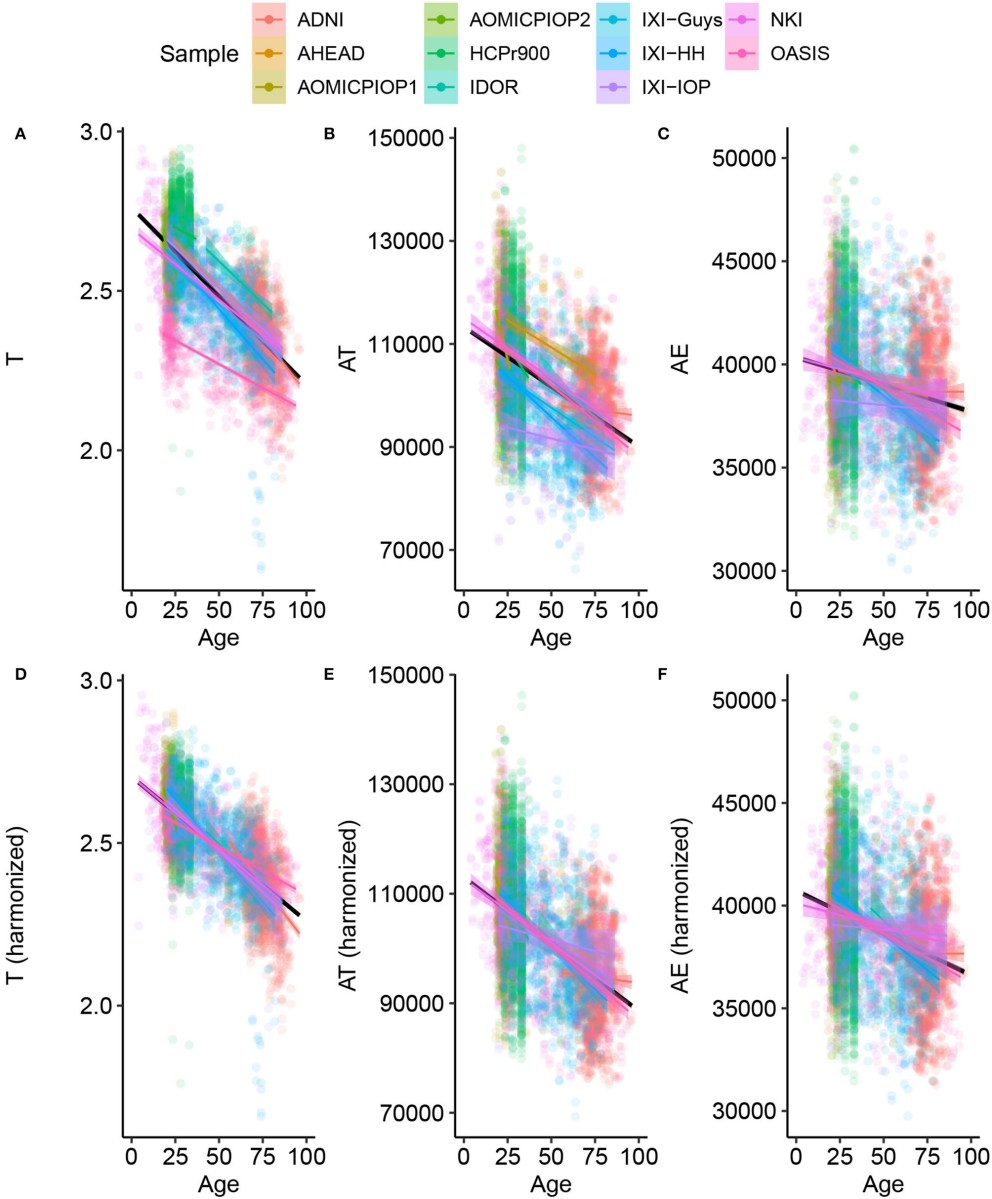
Basic morphological variables through age for Healthy Controls **(A–C)** raw data **(C–F)** after harmonizing, removing the estimated residual from the Linear Mixed Model. This procedure results in the harmonized values of the novel morphological variables K, S, and I.

The analysis starts with data harmonization, a crucial step to combining information from multiple sites allowing a fair comparison among datasets. Figure 1 shows the result of the harmonization procedure and its reflection on the primary morphological variables. The LMMs are used to fit the data assuming a single angular coefficient for all samples and different linear coefficients due to the uncertainty caused by systematic effect. We considered the variables' trend with age linear based on the correlation between *T*, *A*_*T*_, and *A*_*E*_ with Age for the Healthy Control group calculated from our data. There was a statistically significant correlation for T (Pearson *r* = −0.6949; *R*^2^ = 0.48; *p* < 0.001), *A*_*T*_ (Pearson *r* = −0.4711; *R*^2^ = 0.22; *p* < 0.001), and *A*_*E*_ (Pearson *r* = −40.2005; *R*^2^ = 0.42; *p* < 0.001). The harmonization was validated as the most probable description of this data using the Bayesian approach.

### 3.2. Baseline, Rate Estimates, and Alzheimer's Disease Diagnostic Discrimination

An immediate consequence of the harmonization is defining a baseline value and its uncertainty for any morphological variable considered in this work. The LMM provides quantitative information about the trends of these variables through age. The estimated uncertainties values are summarized in Table 2, and summaries of linear mixed models are available in Supplementary Material. The so-called Natural Fluctuation (σ_Natural_), representing human diversity, was considered the Residual Standard Deviation of the models. The approximation for the random component (σ_Random_) of the experimental error derived from the multiple-image acquisition and processing obtained using only 50 subjects from the AOMIC ID1000 dataset is fully described in Supplementary Material. We considered the Random Effect of the Sample category (intercept) Standard Deviation as the uncertainty caused by a systematic effect component of the experimental error (σ_Sample_). The σ_*X*_ is the resultant uncertainty, composed of the previously described errors, and is the square root sum of each one's squared values. Thereby, σ_*K*_ = 0.026 (4.9%), σ_*S*_ = 0.14 (1.6%), and σ_*I*_ = 0.091 (0.9%) with the corresponding fraction of their healthy controls intercept.

**Table 2:** Summary of the estimated uncertainty components for each morphological variable of interest.

**Variable**	**Natural fluctuation**	**σ_*acquisition*_+σ_*processing*_**		** $\sigma_{X}=\sqrt{\Sigma \sigma_{i}^{2}}$ **
	**σ_*Natural*_**	**σ_*Random*_*^a^***	**σ_*Sample*_**	
T [mm]	0.1	0.019	0.085	0.13 (5.35%)
*A*_*T*_ [mm^2^]	9,700	290	4900	10871.3 (10.78%)
*A*_*E*_ [mm^2^]	2,900	70	530	2948.86 (7.62%)
GI	0.095	0.0069	0.11	0.14 (5.60%)
K	0.016	0.0017	0.021	0.026 (4.94%)
S	0.12	0.014	0.076	0.14 (1.56%)
I	0.082	0.0064	0.037	0.091 (0.87%)

a *Repeat measure, mean standard deviation*.

**Figure 2:**
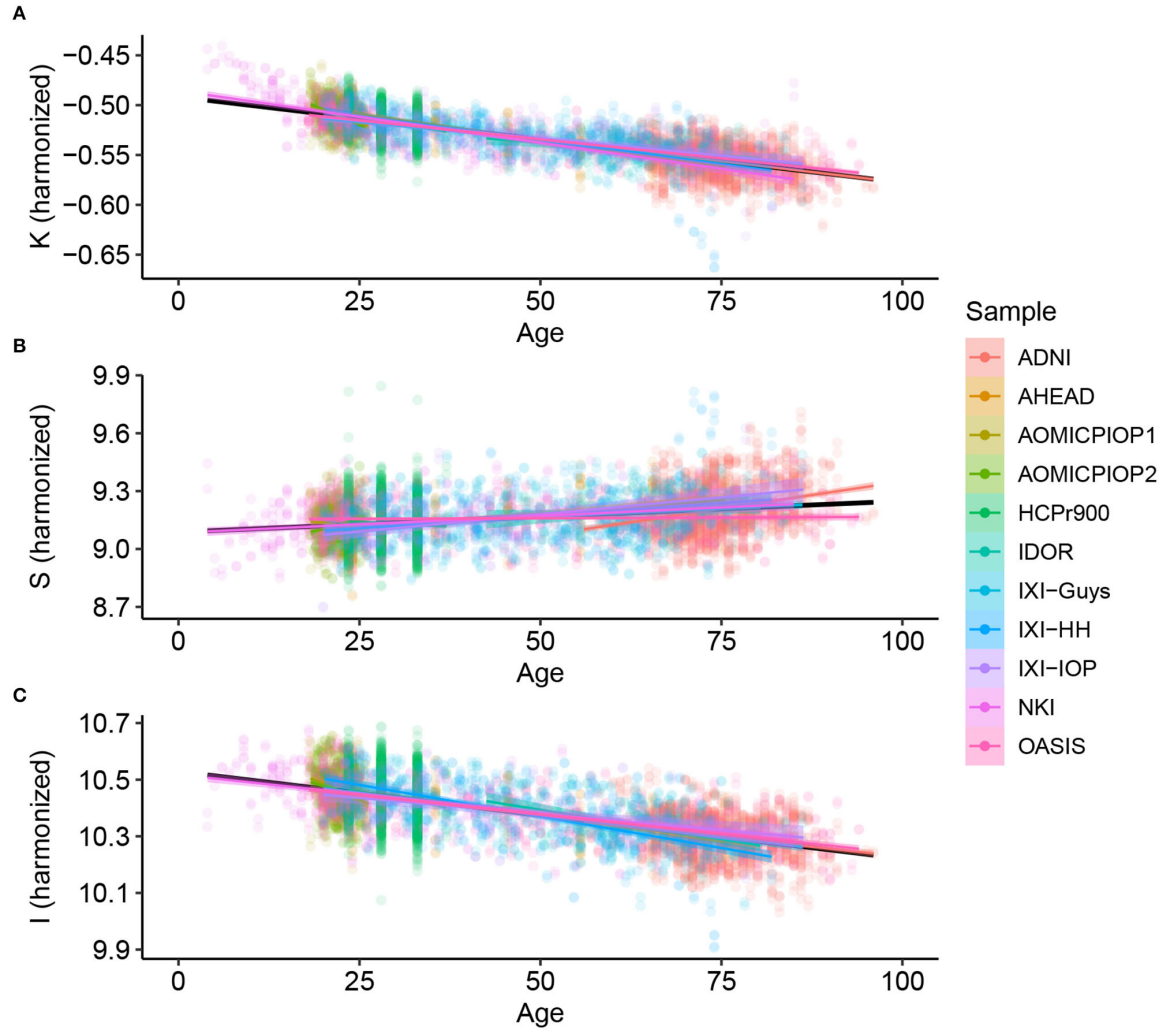
Cortical folding variables **(A)** K, **(B)** S, and **(C)** I through age for Healthy Controls after harmonizing in the primary morphological variables T, AT , and AE (removing the estimated residual from the Linear Mixed Model).

**Figure 3:**
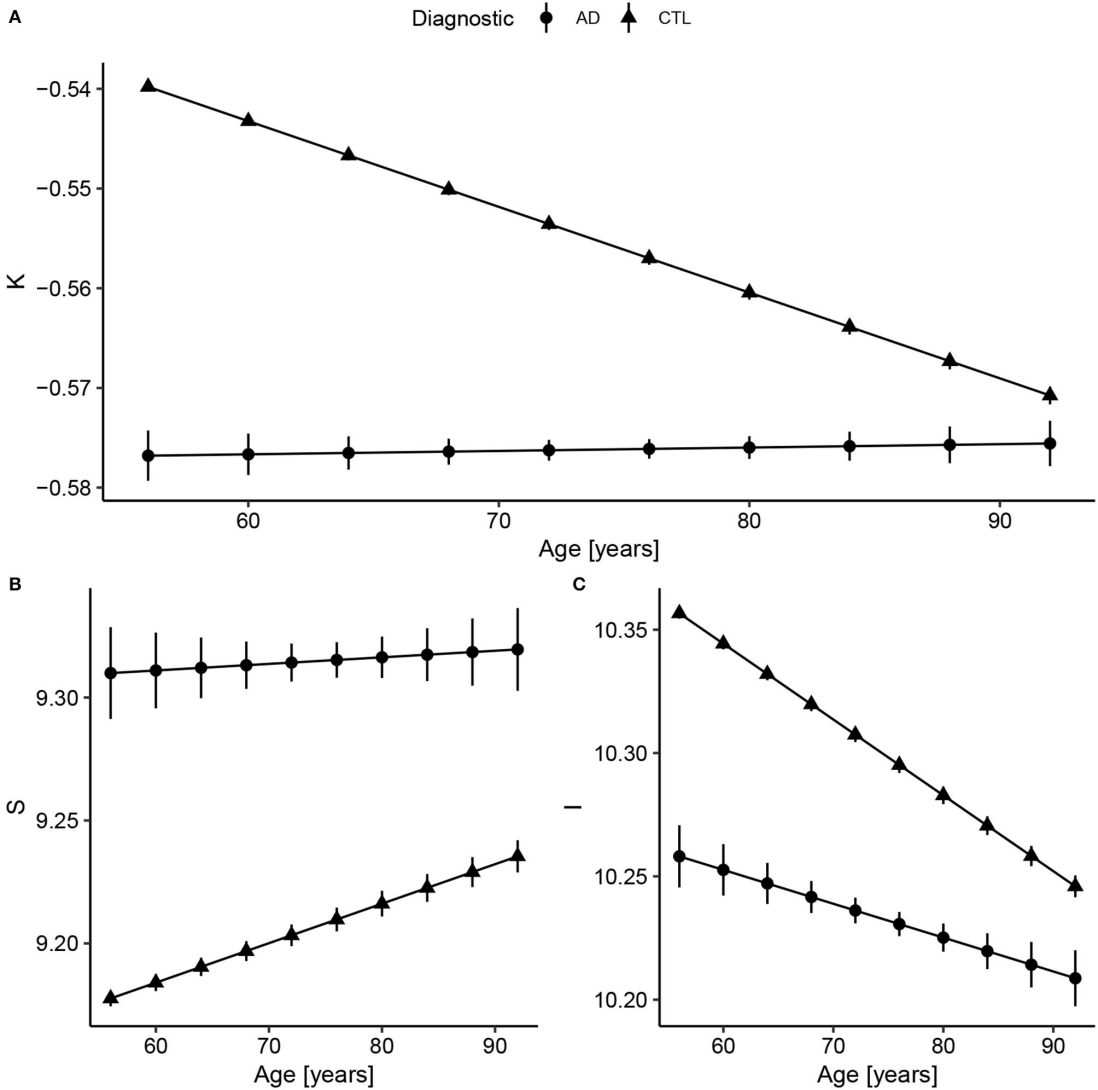
Fitted values extracted from the linear regression model after data harmonization. Bars represent the 95% CI. Complete summary of linear models in Supplementary Material. **(A)** Concerning K, Alzheimer's Disease (AD) has a shallow slope, meaning small changes with Age. Compared to healthy controls, AD values of K are almost constant and similar to older subjects (AD slope *p* < 0.0001 and CTL slope *p* < 0.0001; pairwise comparison estimate −0.00090, *p* < 0.001). **(B)** For S, the AD and CTL patterns have similar intercepts, but statistically different slopes (AD slope *p* = 0.004 and CTL slope *p* < 0.0001; pairwise comparison estimate 0.0013, *p* = 0.004). **(C)** For I, that reflects brain volume, CTL has a decreasing volume with aging, while AD has a smaller slope (AD slope *p* < 0.0001 and CTL slope *p* < 0.0001; pairwise comparison estimate −0.0017, *p* < 0.001). The curves for the AD group were extended to early ages to ease the comparison.

Considering Alzheimer's Disease, we used the baseline (Figure 2) to verify if the age trends of K, S, and I are different between the diseased and Healthy Control groups. We extracted the rates of change for each variable and diagnostic, summarized in Table 3 and displayed in Figure 3. The slopes were compared with *post-hoc* pairwise mean comparisons.

**Table 3:** Changing rate per year for each variable (after harmonization) and diagnostic.

**Diagnostic**	**CTL**	**AD**
T [mm]	(−440 ± 6) × 10^−5^ mm/year (−0.18%)	(−14 ± 4) × 10^−4^ mm/year(−0.062%)
*A*_*T*_ [mm^2^]	(−240 ± 5) mm^2^/year (−0.24%)	(−9. ± 4) × 10 mm/year (-0.096%)
*A*_*E*_ [mm^2^]	(−41 ± 1) mm^2^/year (−0.11%)	(−4 ± 1) × 10 mm/year (−0.1%)
GI	(−350 ± 5) × 10^−5^/year (−0.13%)	(3 ± 4) × 10^−4^/year (0.012%)
K	(−860 ± 9) × 10^−6^/year (−0.16%)	(3 ± 6) × 10^−5^/year (0.0059%)
S	(160 ± 6) × 10^−5^/year (0.018%)	(3 ± 5) × 10^−4^/year (0.0029%)
I	(−310 ± 4) × 10^−5^/year (−0.03%)	(−14 ± 3) × 10^−4^/year (−0.013%)

*Rate [%] was calculated as the fraction of the mean value*.

*Mean value ± SD. Rate [%] was estimated as the fraction of the mean value*.

There is a significant difference between Control and AD slopes in K (pairwise comparison estimate −0.00090, *p* < 0.001), S (pairwise comparison estimate 0.0013, *p* = 0.004), and I (pairwise comparison estimate −0.0017, *p* < 0.001) as expected from Figure 3. For K, the AD curve is virtually flat as if it had reached a plateau, while for I, we see a constant reduction in brain isometric volume, with a reduced intercept. Comparing the mean values within AD and CTL, there is a significant difference for K (pairwise comparison estimate 0.042, *p* < 0.001, representing 7.9% of the CTL mean) value, S (pairwise comparison estimate −0.14, *p* < 0.001, 1.5%), and I (pairwise comparison estimate 0.11, *p* < 0.001, 1.0%).

**Figure 4:**
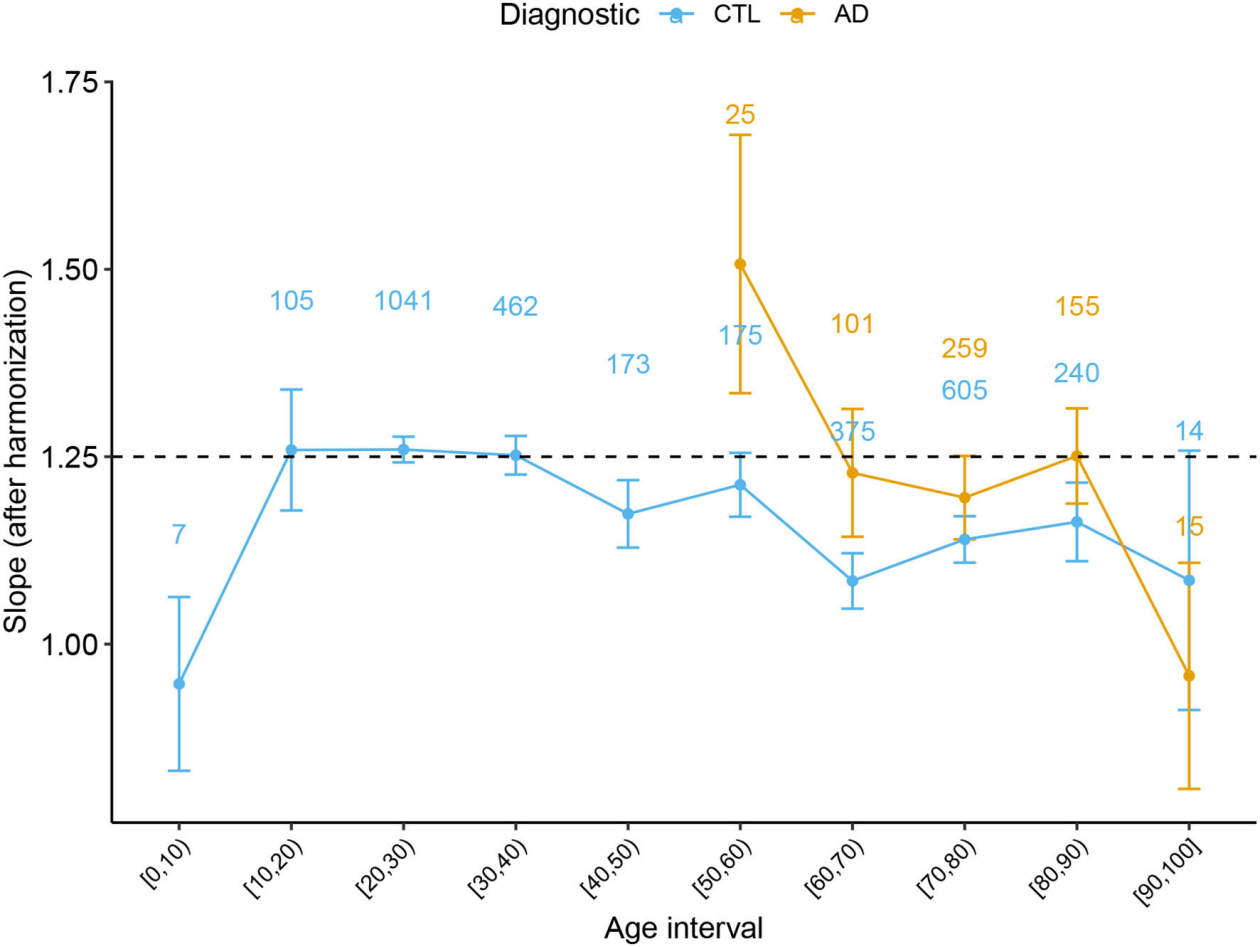
Slope α with 95% CI derived from the Cortical Folding model from Mota and Herculano-Houzel through age for Healthy Controls and AD subjects. Data points from (0, 5] and [95, 100] years old were omitted due to the reduced data. The numbers on top of each point indicate the number of subjects at each interval.

In order to complete the full description of the cortical morphology within the framework established by the Mota and Herculano-Houzel's model, we separated the data from both Health controls and patients with AD per decade and fitted the self-similarity dimension α theorized to be 1.25. The result is shown in Figure 4.

As most diseases imply local or non-uniform global structural damage, we expanded the results of trajectories to the lobes. We included ROI as a fixed effect in the linear mixed model equation with harmonized variables: *Variable*~*Age*×*Diagnostic*×*ROI*+(1|*Sample*:*Diagnostic*:*ROI*). Rates are summarized in Supplementary Material. We discriminated AD and healthy aging (CTL) K trends with pairwise comparisons at the Frontal (*p* adj <0.0001), Occipital (*p* adj <0.0001), Parietal (*p* adj <0.0001), and Temporal lobes (*p* adj <0.0001), as expected from de Moraes et al. (2022). Despite the rates being smaller for AD than CTL, the results mimic the whole brain with Alzheimer's Disease trajectories appearing as a plateau during aging. The Frontal and Parietal lobe shows the least folded pattern (K) in AD (within lobe comparison are described in Supplementary Material). Also, there are significant differences between S pairwise comparisons in CTL and AD at the Frontal (*p* adj = 0.00035) and Parietal lobes (*p* adj <0.0001); and among I, between CTL and AD at the Frontal (*p* adj <0.0001), Occipital (*p* adj = 0.0031), Parietal (*p* adj <0.0001), and Temporal lobes (*p* adj <0.0001)).

## 4. Discussion

We analyzed a combination of datasets aiming to establish the K, S, and I baseline through the human lifespan. The suggested model included complexity at two levels: accounting for heterogeneous acquisition and processing and heterogeneous brain structures across healthy and disease subjects. The hypothesis is that non-homogeneous methodology (inclusion criteria, acquisition, and processing) implies more significant uncertainty due to a systematic effect. Thereby, we suggested a harmonization procedure that allows the comparison of data from multisite reducing this kind of experimental error. This result is innovative in its simplicity and focuses on expanding our independent morphological variables knowledge. The main caveat of our procedure is the impossibility of accounting for all differences between the samples and using this to define a universal harmonization according to each acquisition protocol. Our method does not depend on one reference sample or a gold standard definition. The removal of the shift for each sample is carried out based on the available healthy control data, which can always be added to the analysis.

In other words, our method does not account for a possible uncertainty due to a global systematic effect that could shift all samples equally. By looking at the uncertainty in cortical thickness estimation, our results are compatible with previous work. Our findings of 0.02 mm (Table 2) of uncertainty in cortical thickness are in agreement with a variability less than 0.03 mm found in a test-retest analysis by Han et al. (2006). Moreover, they suggest that uncertainties of 0.15 mm across platforms of manufacturers and 0.17 mm across field strength, at this study, coupled with processing uncertainties and estimated in 0.14 mm. A most recent study by Frangou et al. (2020) suggests a Mean Inter-individual variation of 0.07 ± 0.06 mm for hemisphere's cortical thickness at all age ranges in the study compared to 0.10 mm from our findings. This compatibility suggests that the possible global systematic effect is close to 0; thus, the harmonization could be regarded as being close to the universal one.

The typical values for the K, S, and I independent variables through the whole life span and their related uncertainties were obtained by this study. These curves not only can be used to discriminate pathological from healthy aging in cross-sectional harmonized data but also are a powerful tool to morphologically describe non-typical cortex. Estimating the uncertainties is crucial to constructing the baseline curves and was carefully developed by this study. We estimated three types of uncertainties with linear mixed models and repeated measures: the uncertainty due to a systematic effect (combination of multiple datasets), repeated measures, and the natural variation in the human species. The error from acquisition and processing were coupled at the individual and sample levels. We expect future studies to disassociate the uncertainties in detailed factors (test-retest analysis, across platforms, field strengths, and acquisition parameters with the same sample of subjects) to measure morphological variables of interest better.

We have demonstrated the usage of the baseline curves by analyzing AD data. In terms of K, a measure of axonal tension, the AD curve is virtually flat as if it had reached a plateau of reduced brain tension. Compared with Healthy Controls, this plateau seems to be the same experienced by the oldest subjects. Interpreting in terms of the Mota and Herculano-Houzel cortex model, AD can be regarded as a form of accelerated aging from the axonal tension perspective. On the other hand, the evolution of S, I, and even the self-similarity dimension α suggest that the AD cortex is not morphologically similar to the health one, with a very different shape, isometric volume, and time evolution. However, it is interesting to note that both S and I converge to a value compatible with the oldest health subjects. In the future, correlating these results with the elasto-mechanic properties of the AD cortex will help to understand better the physical limits of the Mota and Herculano-Houzel model and expand it theoretically.

A significant result that could only be achieved by combining multiple data is the value of the self-similarity dimension α through the human lifespan and its evolution through atypical conditions. We extended the analysis in de Moraes et al. (2021) and verified how the slope α behaves with healthy aging. We found that the slope is compatible with the theoretical prediction for subjects between 20 and 60 years old. Defining this range of applicability of the theory is essential to understanding the period of life in which the model's basic assumptions are still valid. Thereafter, we confirmed the findings that the slope escapes the model after 60 years old, diverging from the theoretical value of 1.25. Before 15 years old, we have found a hint that the slope is also inferior to 1.25, in disagreement with the model. In addition, a non-linear pattern is suggested by the K baseline function before 20 years. One of the leading hypotheses to explain the deviation of the measured α from the expected value in both cases (before 15 and after 60 years old) is the breakdown of the homogeneity on the cortical surface. The suggestion of a homogeneity breakdown makes it particularly interesting to further study AD in the age range compatible with 1.25. Understanding the regimes outside the model is a fundamental step toward the theoretical understanding of cortical folding. Coupled with analysis of neuropathologies such as AD, these empirical results improve the cortical folding theory.

More than discriminating between disease and healthy subjects, understanding the pathology trajectory is vital to defining landmarks, indicating future injury, and collaborating to develop efficient treatment. Notably, in Alzheimer's Disease and its prodromal form, Mild Cognitive Impairment, subsequent phases of damage may start at least 20 years before the diagnosis (Frozza et al., 2018). The structural damage is an initial state than cognitive alterations, such as memory and clinical functions (Jack et al., 2010b). As pointed out by Fjell et al. (2014), healthy aging also contributes to reductions in cortical thickness and understanding the overlap between healthy aging and pathological aging triggers is crucial to segregating both events. Nevertheless, the possible similarity of structural damage in Alzheimer's Disease and aging is explored by multiple previous studies (Pacheco et al., 2015; Gutierrez Becker et al., 2018; Huizinga et al., 2018; Wang et al., 2021). We hypothesize that the structural changes due to AD are reflected in measures of the brain's global structure and shape. Therefore, cortical folding derived variables K, S, and I are sensitive to this perturbation. We verified this hypothesis by analyzing the difference in aging trajectories between healthy and pathological brains.

At a more refined grain scale, the lobes, the results are compelling to confirm (Wang et al., 2021) suggestion that K, S, and I together would be a powerful tool to discriminate similarly but distinct events, such as AD and aging. In this case, the difference between events is in the cortical structure's changing velocity or rate. Then with the rates for K, S, and I at the lobes, we could explore whether a lobe would unfold faster. For AD, the temporal lobe has the biggest unfolding (K) and shrinking rate (I), while healthy aging is more aggressive in the Parietal lobe, followed by the temporal and frontal lobes.

The natural expansion of this study is to include Mild Cognitive Impairment (MCI) subjects. As seen before, the MCI is an intermediary step between healthy aging and AD in the disease progression, cognitive impairment, and structural changes (Jack et al., 2010b; Frozza et al., 2018; de Moraes et al., 2022). Also, we intend to continue this study by applying this approach to a longitudinal dataset composed of MRI structural images with MCI subjects that eventually convert to AD and compare to the cortical folding components of non-converters. Including this data would allow having a more reasonable aging trajectory by having the transition period from healthy aging to pathological aging. Furthermore, we expect to explore K, S, and I function with age for the age range comprising newborns to 25 years old in future studies. This study will help better understand the value of the slope α at this age, revealing if there is an inflection point that can be connected to the development/aging transition in human brains (Davis, 2021), such as suggested by the data.

This study successfully achieved its goal of combining multiple samples with a simplistic harmonization procedure that adjusts K, S, and I values for the 3,650 subjects. Hereafter we estimated K, S, and I typical values and aging rates to discriminate against Alzheimer's Disease subjects based on their changing rates. To validate the proposed methodology, we analyzed results for Cortical Thickness, which are in concordance with previously published findings. At a glance, the significant differences found in aging rates for the cross-sectional data are suggestive that a brain with Alzheimer's Disease has premature and accelerated aging in terms of morphology.

## Data Availability Statement

The original contributions presented in the study are publicly available. Data from previous publications are available from their repositories: Wang et al. (2016, 2019a). Data from AHEAD (Alkemade et al., 2020) and AOMIC (Snoek et al., 2021) datasets with the cortical folding variables extracted by these authors are available at https://doi.org/10.5281/zenodo.5750619 (de Moraes et al., 2021). Data acquired at IDOR does not have clearance for public sharing patients' information that could lead to identification due to local Ethics Committee approval restrictions but will be shared upon request.

## Ethics Statement

The studies involving human participants were reviewed and approved by Copa D'Or Research Ethics Committee under the protocol number CAAE 47163715.0.0000.5249. Written informed consent to participate in this study was provided by the participants' legal guardian/next of kin.

## Author Contributions

FM, VM, FT-M, and BM contributed to the conception and design of the study. FM organized the database. FM and VM performed the statistical analysis and wrote the first draft of the manuscript. FT-M and BM supervised and were responsible for the project administration and funding acquisition. All authors contributed to the article and approved the submitted version.

## Funding

This study was funded by the Research Support Foundation of the State of Rio de Janeiro (FAPERJ), National Council for Scientific and Technological Development (CNPq), and intramural grants from Instituto D'Or de Pesquisa e Ensino (IDOR). BM was supported by Instituto Serrapilheira (Grant Serra-1709-16981) and CNPq (PQ 2017 312837/2017-8). ADNI data included in this work collection and sharing was funded by the Alzheimer's Disease Neuroimaging Initiative (ADNI) (National Institutes of Health Grant U01 AG024904) and DOD ADNI (Department of Defense award number W81XWH-12-2-0012). ADNI is funded by the National Institute on Aging, the National Institute of Biomedical Imaging and Bioengineering, and through generous contributions from the following: AbbVie, Azheimer's Association; Alzheimer's Drug Discovery Foundation; Araclon Biotech; BioClinica, Inc.; Biogen; Bristol-Myers Squibb Company; CereSpir, Inc.; Cogstate; Eisai Inc.; Elan Pharmaceuticals, Inc.; Eli Lilly and Company; EuroImmun; F. Hoffmann-La Roche Ltd and its affiliated company Genentech, Inc.; Fujirebio; GE Healthcare; IXICO Ltd.; Janssen Alzheimer Immunotherapy Research & Development, LLC.; Johnson & Johnson Pharmaceutical Research & Development LLC.; Lumosity; Lundbeck; Merck & Co., Inc.; Meso Scale Diagnostics, LLC.; NeuroRx Research; Neurotrack Technologies; Novartis Pharmaceuticals Corporation; Pfizer Inc.; Piramal Imaging; Servier; Takeda Pharmaceutical Company; and Transition Therapeutics. The Canadian Institutes of Health Research is providing funds to support ADNI clinical sites in Canada. Private sector contributions are facilitated by the Foundation for the National Institutes of Health (www.fnih.org). The grantee organization is the Northern California Institute for Research and Education, and the study is coordinated by the Alzheimer's Therapeutic Research Institute at the University of Southern California. ADNI data are disseminated by the Laboratory for Neuro Imaging at the University of Southern California.

## Conflict of Interest

The authors declare that the research was conducted in the absence of any commercial or financial relationships that could be construed as a potential conflict of interest.

## Publisher's Note

All claims expressed in this article are solely those of the authors and do not necessarily represent those of their affiliated organizations, or those of the publisher, the editors and the reviewers. Any product that may be evaluated in this article, or claim that may be made by its manufacturer, is not guaranteed or endorsed by the publisher.

## Footnotes

^1^One may understand this as similar to “Principal Component Analysis”, a change of basis on the data representation, but using scaling considerations rather than covariance eigenvectors to define the principal directions. The new set of variables are combinations of the usual geometrical variables used to describe the cortex. The principal component K is given by the power-law relation, which contains the correlation between the variables. The variables S and I are orthonormal to K.

^2^One can also start requiring a plane perpendicular to *K*, impose the dimensionless requirement, resulting in the dimensionless vector *S*, and find *I* from *K*×*S*.

^3^“Similar to batch effects in genomics (see Leek et al. (2010) for a review of batch effects), we use the term scanner effects in neuroimaging to refer to unwanted variation that is (1) non-biological in nature and (2) associated with differential scanning equipment or parameter configurations. Because different imaging sites use different physical scanners, site effects are one example of scanner effects”.
