# Supplementary material for: Establishing a Baseline for Human Cortical Folding Morphological Variables: A Multisite Study
Source: Front Neurosci. 2022 Jul 18;16:897226. doi: 10.3389/fnins.2022.897226 (PMC9340792; doi:10.3389/fnins.2022.897226)
Supplement: Supplementary file 1 [file Presentation_1.pdf]

Supplementary Material

1 SUPPLEMENTARY INFORMATION, TABLES, FIGURES AND RESULTS

Part of the data used in preparation of this article were obtained from the Alzheimer's Disease Neuroimaging Initiative (ADNI) database (adni.loni.usc.edu). As such, the investigators within the ADNI contributed to the design and implementation of ADNI and/or provided data but did not participate in analysis or writing of this report. A complete listing of ADNI investigators can be found at: `http://adni.loni.usc.edu/wp-content/uploads/how_to_apply/ADNI_Acknowledgement_List.pdf`. The ADNI was launched in 2003 as a public-private partnership, led by Principal Investigator Michael W. Weiner, MD. The primary goal of ADNI has been to test whether serial magnetic resonance imaging (MRI), positron emission tomography (PET), other biological markers, and clinical and neuropsychological assessment can be combined to measure the progression of mild cognitive impairment (MCI) and early Alzheimer's disease (AD).

Table S1. Summary for each dataset. Mean value ± standard deviation. Cross-sectional analyses were performed compilating all samples into one heterogeneous dataset.

Datasets	Diagnostic	N (F)	Age [years]	MRI equipment	FreeSurfer
ADNI Jack et al. (2010)	CTL	868 (445)	75 ± 6.5	multiple 3T	v5.3
	AD	542 (241)	75 ± 8		
AHEAD[1] Alkemade et al. (2020)	CTL	100 (56)	42 ± 19	Philips Achieva 7T	v6
AOMIC PIOP01 Snoek et al. (2021)	CTL	208 (120)	22 ± 1.8	Philips Achieva 3T	
AOMIC PIOP02 Snoek et al. (2021)	CTL	224 (128)	22 ± 1.8	Philips Achieva dStream 3T	
AOMIC ID1000 Snoek et al. (2021)	CTL	50 (27)	23 ± 1.7	Philips Achieva dStream 3T	
HCP900r[1] Glasser et al. (2013)	CTL	881 (494)	29 ± 3.6	Siemens Skyra (modified) 3T	v5.3[2]
IDOR de Moraes et al. (2022)	CTL	77 (53)	66 ± 8.4	Philips Achieva 3T	v6
	AD	13 (8)	77 ± 6.1		
IXI-Guy's[3,4]	CTL	314 (175)	51 ± 16	Philips Intera 3T	v5.3
IXI-HH[4]	CTL	181 (94)	47 ± 17	Phillips Gyroscan Intera 1.5T	v5.3
IXI-IOP[6]	CTL	68 (44)	42 ± 17	GE 1.5T	v5.3
NKI Nooner et al. (2012)	CTL	168 (68)	34 ± 19	Siemens Magnetom 3T	v5
OASIS[7] Marcus et al. (2010)	CTL	312 (196)	45 ± 24	Siemens Vision Scanner 1.5T	Dev 20061005

Diagnostic: CTL – Healthy control; AD – Alzheimer's Disease
[1]AHEAD and HCPr900 datasets provided an age range for each subject. Therefore, we supposed that each subject's age was the provided interval's mean age.
[2]modified version
[3]http://brain-development.org/ixi-dataset/
[4]IXI subset: Guy's Hospital
[5]IXI subset: Hammersmith Hospital
[6]IXI subset: Institute of Psychiatry
[7]Data were only available for hemispheres analysis, not used for the lobes tests.

Table S2. Linear mixed models summary. Raw data, with no harmonization. 4364 observations; 11 Diagnostic:Sample; 8 Sample.
Equation: $Variable \sim Age \times Diagnostic + (1|Sample : Diagnostic)$.

Variable	Residual SE		R^2		Effect	Estimate	Std. Error	t value	Pr(—t—)	
	Data	Diagnostic:Sample (Intercept)	Marginal	Conditional						
T	0.1049504	0.0847818	0.02454712	0.461506	0.684592	(Intercept)	2.70	0.027	99.87	0.00E+00
						Age	-0.0044	0.00011	-41.19	0.00E+00
						AD	-0.34	0.041	-8.43	0.00E+00
						Age:AD	0.0030	0.00041	7.31	2.77E-13
K	0.0163037	0.0210008	0.002146375	0.437232	0.789741	(Intercept)	-0.49	6.41E-03	-76.73	0.00E+00
						Age	-0.00086	1.68E-05	-51.40	0.00E+00
						AD	-0.084	5.44E-03	-15.50	0.00E+00
						Age:AD	0.00089	6.37E-05	13.91	0.00E+00
S	0.1215955	0.0761271	0.01779927	0.151572	0.399721	(Intercept)	9.084	2.43E-02	374.38	0.00E+00
						Age	0.0017	1.25E-04	13.66	0.00E+00
						AD	0.20	4.13E-02	4.77	1.88E-06
						Age:AD	-0.0014	4.75E-04	-2.92	3.46E-03
I	0.0818765	0.0373436	0.01363622	0.450591	0.555409	(Intercept)	10.53	1.26E-02	836.069	0.00E+00
						Age	-0.0031	8.37E-05	-37.25	0.00E+00
						AD	-0.18	2.85E-02	-6.42	1.35E-10
						Age:AD	0.0017	3.20E-04	5.47	4.59E-08

2 UNCERTAINTIES FROM REPEATED MEASURES

The random error is an essential fraction of the global uncertainties, as it is the variance that occurred after multiple measures in the same condition. To estimate the reproducibility within/subjects, we processed (as described in Materials and Methods) the first 50 subjects with all three T1w images from the AOMIC ID1000 Snoek et al. (2021) dataset. It should be emphasized that this random error is only an approximation as we are analyzing only one sample. For a proper description of this uncertainty, one would need a diligent procedure to track all differences between acquisitions as well as multiple images of the same individual for each sample, a huge experimental effort yet to be done. The AOMIC ID1000 comprises multiple Magnetic Resonance Imaging protocols, especially three structural T1w images acquired during the same scan with the same protocol in a Philips Intera 3T. The select sample has the advantage of being composed of only healthy human subjects within a narrow age range, from 20 to 26 years old.

To estimate the uncertainty/variation of repeated measures, one could estimate the distribution of standard deviations of the data. We calculated the standard deviation for each subject and hemisphere across the three images, for the Cortical Thickness, K, S, and I. Then, we compared the means for each run with a Repeat Measure ANOVA.

We hypothesized that should be no difference in means within the three runs, despite the variation for each subject (Figure S1).

We compared the means for each run with a Repeat Measure ANOVA. There is no significant difference of means through runs (1, 2 and 3) (Figure S2): Cortical Thickness [mm], F(2, 198) = 0.48, p = 0.62, eta2[g] = 0.00019; GI, F(2, 186) = 0.2, p = 0.81, eta2[g] = 0.00001; K, F(2, 198) = 0.14, p = 0.87, eta2[g] = 0.000014; S, F(2, 185) = 0.35, p = 0.69, eta2[g] = 0.00008; I, F(2, 198) = 0.77, p = 0.46, eta2[g] = 0.000052.

The estimate standard deviations are: Cortical Thickness [mm] (T), 0.019 $\pm$ 0.013 mm; GI, 0.0069 $\pm$ 0.0047; K, 0.0017 $\pm$ 0.0012; S, 0.014 $\pm$ 0.01; I, 0.0064 $\pm$ 0.0043 (Figure S3).

These results suggest high reliability in subsequently acquired images processed with FreeSurfer v6.0, in concordance with Eggert et al. (2012) finds for reliability in FreeSurfer processing comparing volumes measurements.

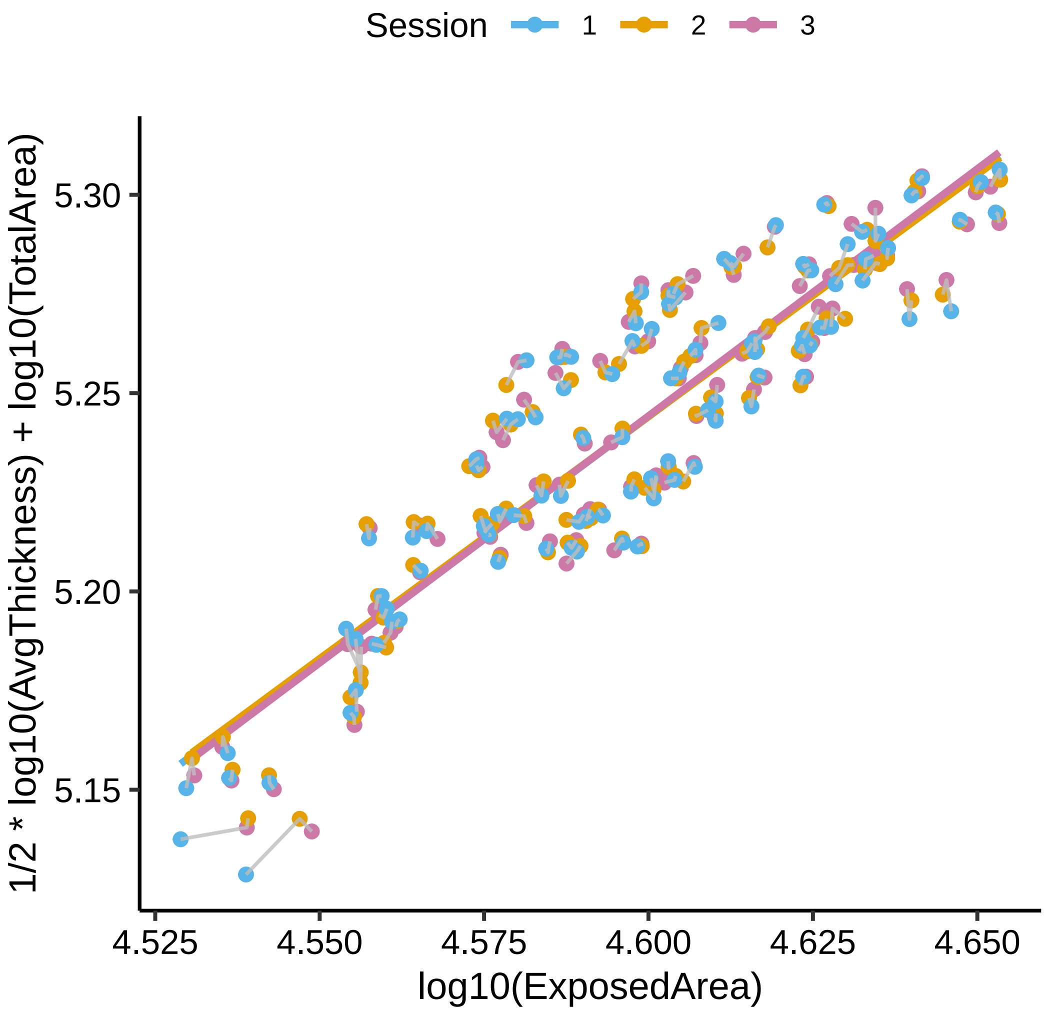

Figure S1. Cortical folding model with the traced path for each hemisphere of each subject (gray line). The sample respects the model with slope $\alpha = 1.23 \pm 0.06$, 95% confidence interval = (1.12,1.34).

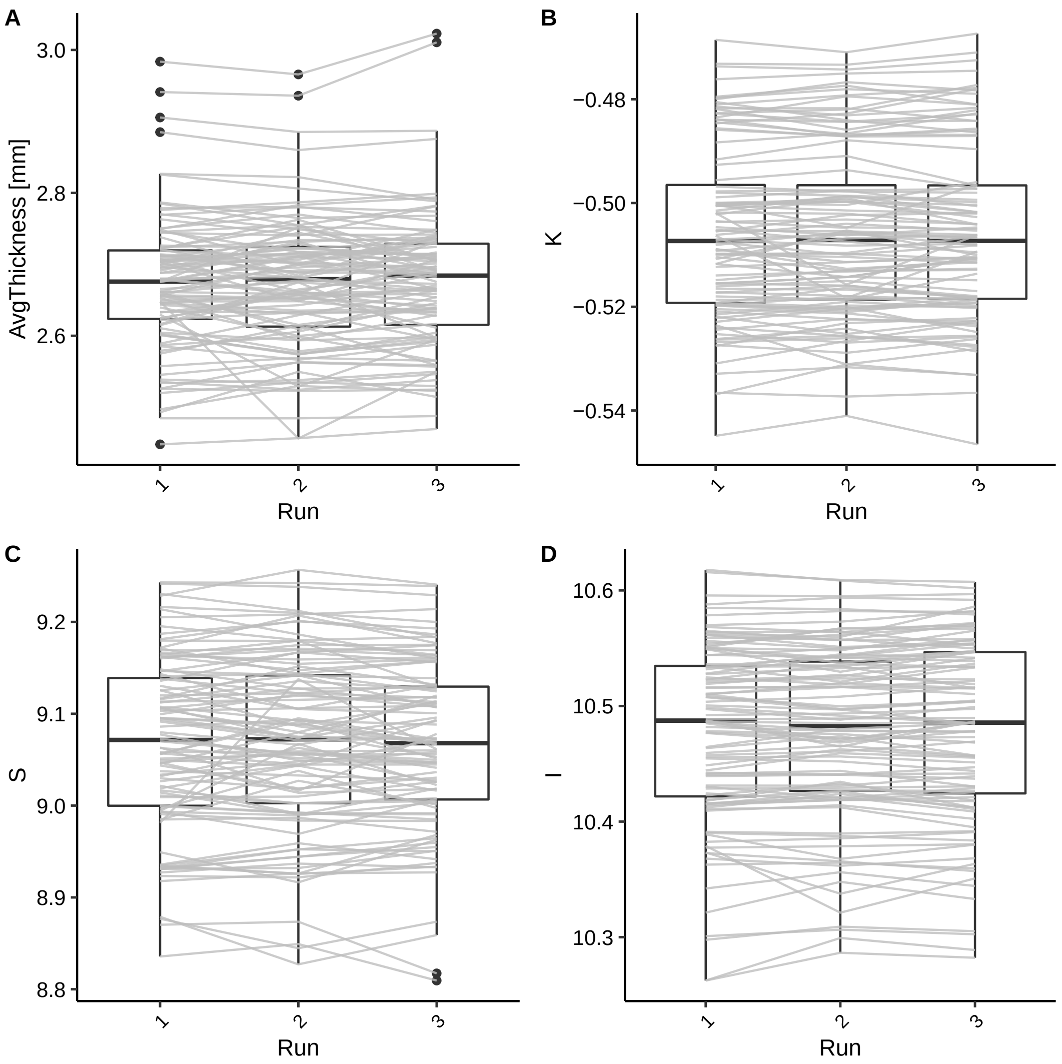

Figure S2. Trajectories for each hemisphere of each subject through the acquisition runs. There is no significant difference in means. (A) Cortical Thickness [mm]: F(2, 198) = 0.48, p = 0.62, eta2[g] = 0.00019; (B) K: F(2, 198) = 0.14, p = 0.87, eta2[g] = 0.000014; (C) S: F(2, 185) = 0.35, p = 0.69, eta2[g] = 0.000080; (D) I: F(2, 198) = 0.77, p = 0.46, eta2[g] = 0.000052.

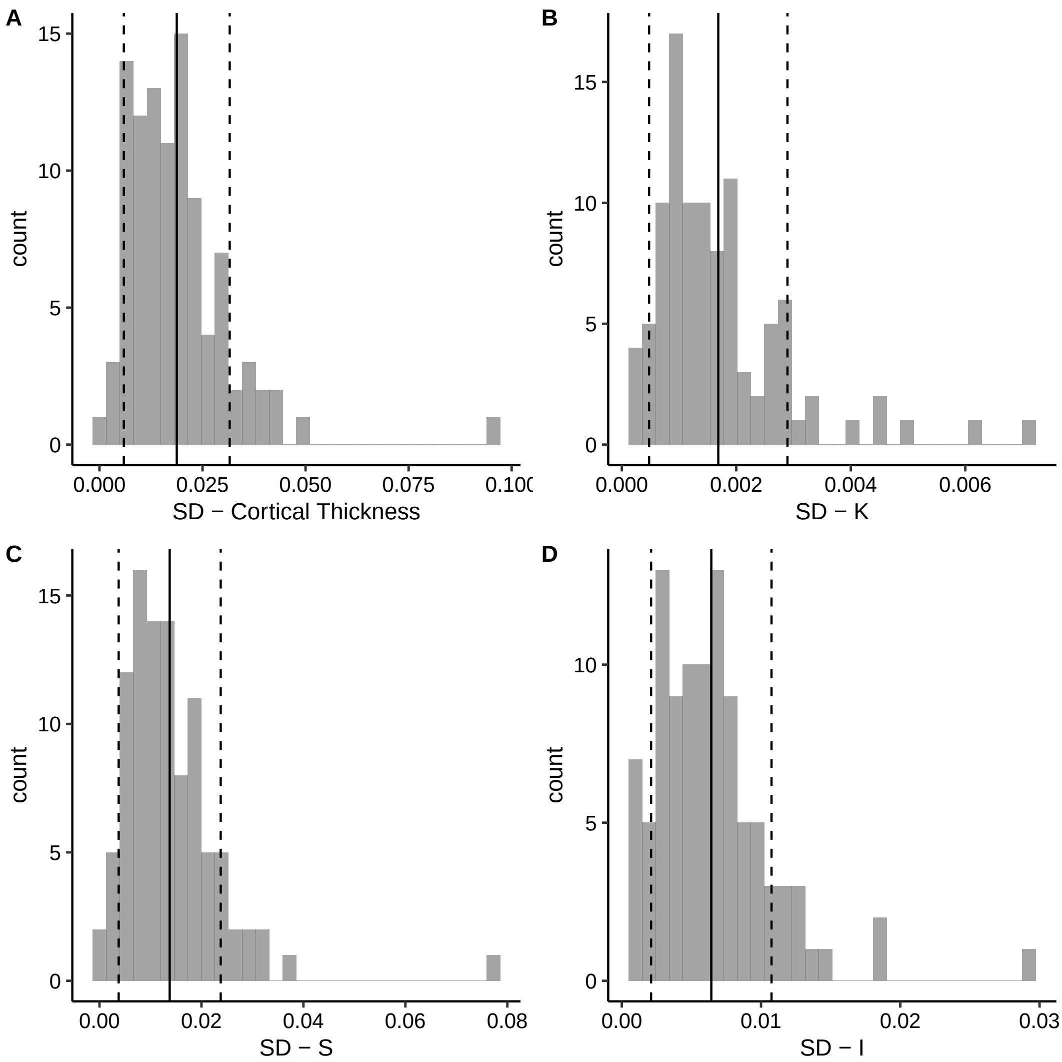

Figure S3. Standard Deviation (SD) distribution for each variable. The solid line represents the mean value, and the dashed line the standard deviation of the distribution. (A) Cortical Thickness: 0.019±0.013 mm; (B) K: 0.0017±0.0012; (C) S: 0.014±0.01; (D) I: 0.0064±0.0043.

3 EXPANDED RESULTS TO LOBES

We expanded the analyses of trajectories to the lobes by including ROI as a fixed effect in the linear mixed model equation (Equation 6 from the main text). Rates are summarized in Table S3 and Figure S4. Differences within lobes and rate differences within diagnostics are described in Figure S5.

Table S3. Changing rate per year for each variable, diagnostic and lobe. Mean value ± standard deviation.

Variable	ROI	CTL	AD
T	F	-0.0058 ± 6.5e-05 (-0.24%)	-0.00095 ± 0.00043 (-0.043%)
	O	-0.0034 ± 6.5e-05 (-0.16%)	-0.0012 ± 0.00043 (-0.06%)
	P	-0.0048 ± 6.5e-05 (-0.21%)	0.0012 ± 0.00043 (0.059%)
	T	-0.0051 ± 6.5e-05 (-0.19%)	-0.0016 ± 0.00043 (-0.067%)
GI	F	-0.0029 ± 6.6e-05 (-0.12%)	0.00038 ± 0.00044 (0.017%)
	O	-0.0025 ± 6.6e-05 (-0.1%)	-0.00049 ± 0.00044 (-0.02%)
	P	-0.0048 ± 6.6e-05 (-0.17%)	0.00026 ± 0.00044 (0.0095%)
	T	-0.0035 ± 6.6e-05 (-0.14%)	-0.00054 ± 0.00044 (-0.023%)
K	F	-0.00092 ± 1.3e-05 (-0.17%)	5.4e-05 ± 8.4e-05 (0.0092%)
	O	-0.00068 ± 1.3e-05 (-0.14%)	-0.00012 ± 8.4e-05 (-0.022%)
	P	-0.0011 ± 1.3e-05 (-0.22%)	0.00031 ± 8.4e-05 (0.053%)
	T	-0.00096 ± 1.3e-05 (-0.18%)	-0.00019 ± 8.4e-05 (-0.033%)
S	F	0.0027 ± 8.2e-05 (0.03%)	0.00027 ± 0.00055 (0.003%)
	O	0.0015 ± 8.2e-05 (0.017%)	-1.1e-05 ± 0.00055 (-0.00013%)
	P	0.0026 ± 8.2e-05 (0.027%)	-0.0023 ± 0.00055 (-0.024%)
	T	0.0022 ± 8.2e-05 (0.025%)	7e-04 ± 0.00055 (0.0078%)
I	F	-0.0036 ± 6e-05 (-0.036%)	-0.00087 ± 4e-04 (-0.0087%)
	O	-0.0029 ± 6e-05 (-0.031%)	-0.0013 ± 4e-04 (-0.014%)
	P	-0.003 ± 6e-05 (-0.029%)	-0.00022 ± 4e-04 (-0.0021%)
	T	-0.003 ± 6e-05 (-0.029%)	-0.00097 ± 4e-04 (-0.0096%)

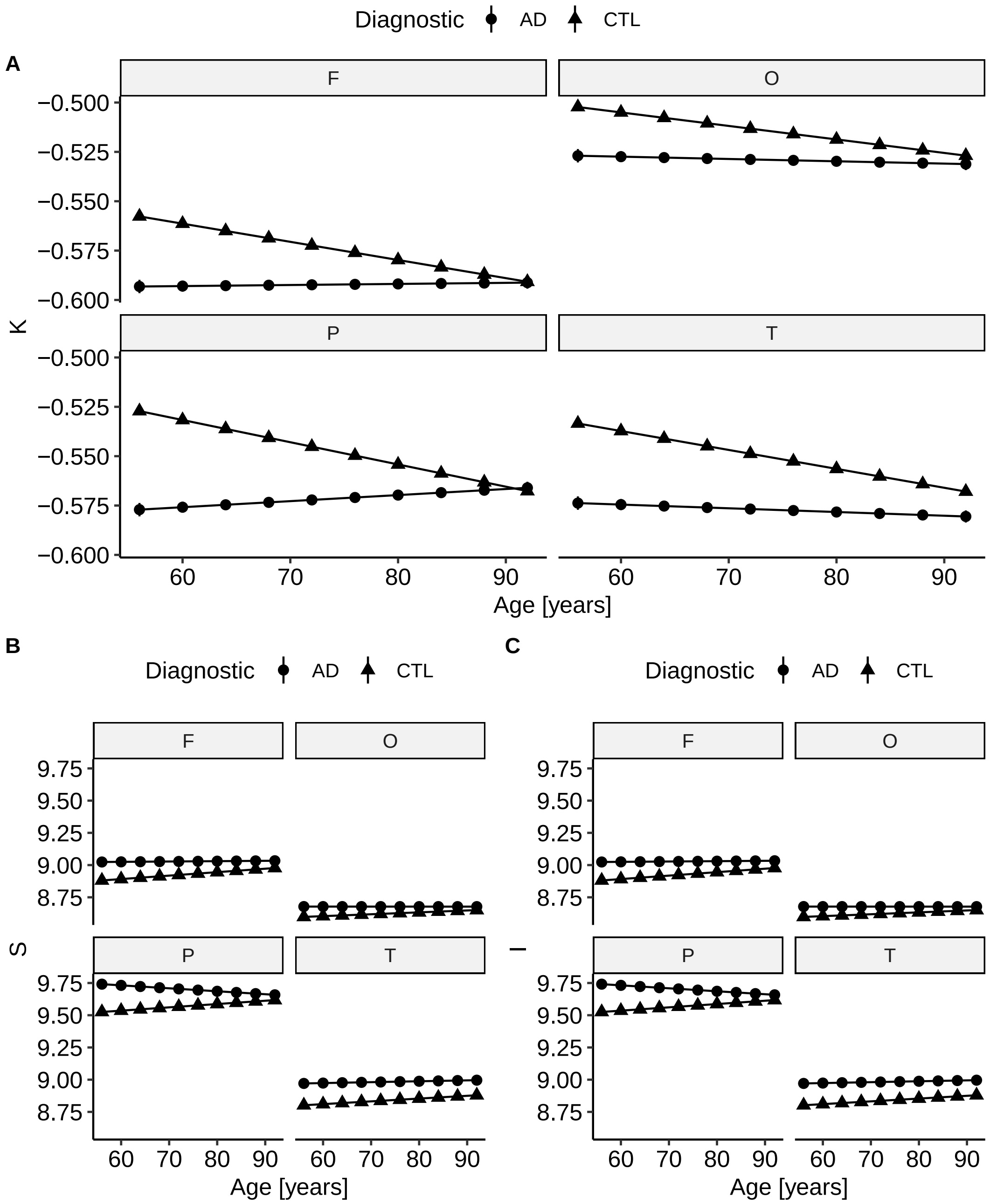

Figure S4. Fitted values for (A) K, (B) S and (I) and age for all for the Frontal lobe ("F"), Parietal lobe ("P"), Occipital lobe ("O") and Temporal lobe ("T") after data harmonization. Bars represents 95% confidence interval.

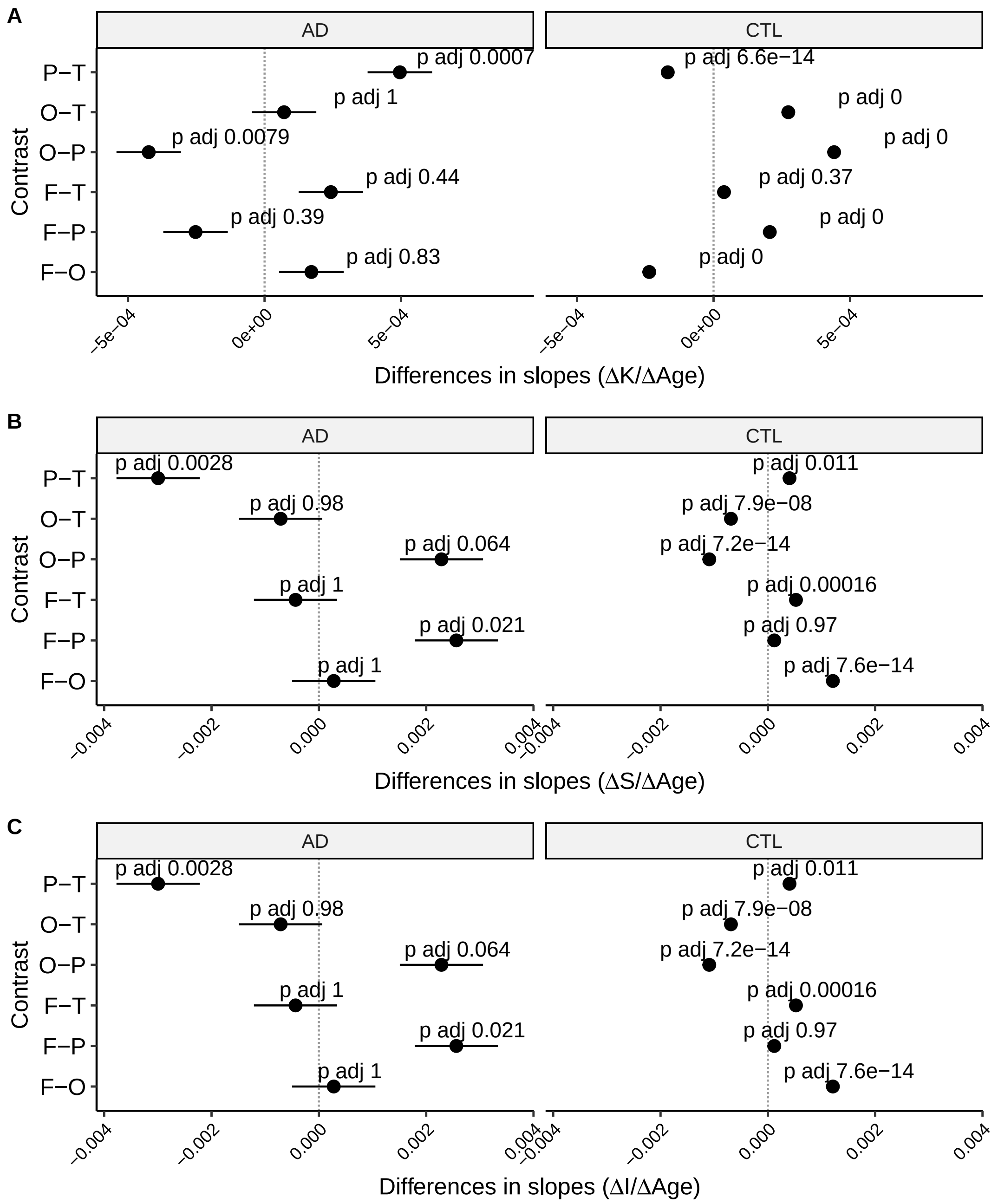

Figure S5. Difference in slopes for each lobe within diagnostics after data harmonization. Bars represent a 95% confidence interval. (A) K, (B) S, and (C) I.

4 MULTICENTER INFLUENCE ON CORTICAL FOLDING MODEL AND ITS INDEPENDENT MORPHOLOGICAL COMPONENTS

We estimated the influence of multicenter datasets in estimating K, S, and I typical values and rates per year across the human lifespan, including as random effects in the linear mixed model. Using these models allows one to calculate the residual variance arising from the methodological heterogeneity and the variance from each category, the different included data samples in this manuscript.

The systematic shifts, estimated from the Linear Mixed Models (LMM, Equation 6), can be used as inputs to multisite harmonization procedures, reducing the influence of heterogeneous methodology (Figure 1). The harmonization consists of subtracting the estimated shift from the raw data of each sample ($X_{harmonized} = X_{raw} - shift_X$). It reduces the systematic error due to the samples, and consecutively, the total uncertainty of the variable (Table S4). A step-by-step code is available de Moraes et al. (2021).

Future studies can progress on the limitation by detailing influences and increasing specificity in methodology, such as the scan field strength, model and manufacturer, and decoupling acquisition imaging processing components.

Table S4. Comparison of estimated error after the multisite harmonization.

Variable	Natural Fluctuation	$\sigma_{acquisition} + \sigma_{processing}$		$\sigma_X = \sqrt{\Sigma\sigma_i^2}$
	($\sigma_{withinspecienaturalvariation}$)	random[1]	systematic (σ_{sample})	
T [mm]	0.1	0.019	0.000	0.10 (4.10%)
A_T [mm^2]	9700	290	0	9704.33 (9.63%)
A_E [mm^2]	2900	70	0	2900.84 (7.5%)
GM volume [mm^3]	33000	3600	0	33195.8 (13.27%)
GI	0.097	0.0069	0.000	0.97 (3.74%)
K	0.017	0.0017	0.000	0.017 (3.19xx%)
S	0.12	0.014	0.000	0.12 (1.32%)
I	0.082	0.0064	0.000	0.082 (0.79%)

[1]repeat measures, mean standard deviation

REFERENCES

Alkemade, A., Mulder, M. J., Groot, J. M., Isaacs, B. R., van Berendonk, N., Lute, N., et al. (2020). The Amsterdam Ultra-high field adult lifespan database (AHEAD): A freely available multimodal 7 Tesla submillimeter magnetic resonance imaging database. *NeuroImage* 221, 117200. doi:10.1016/j.neuroimage.2020.117200

[Dataset] de Moraes, F. H. P., Mello, V. B. B., and Mota, B. (2021). Deaging and harmonization - Cortical Folding in Humans v1.1. doi:10.5281/zenodo.5348575

de Moraes, F. H. P., Sudo, F., Carneiro Monteiro, M., R. P. de Melo, B., Mattos, P., Mota, B., et al. (2022). Independent morphological variables correlate with Aging, Mild Cognitive Impairment, and Alzheimer's Disease. *medRxiv* , 2022.01.10.22268812doi:10.1101/2022.01.10.22268812

Eggert, L. D., Sommer, J., Jansen, A., Kircher, T., and Konrad, C. (2012). Accuracy and Reliability of Automated Gray Matter Segmentation Pathways on Real and Simulated Structural Magnetic Resonance Images of the Human Brain. *PLOS ONE* 7, e45081. doi:10.1371/journal.pone.0045081. Publisher: Public Library of Science

Glasser, M. F., Sotiropoulos, S. N., Wilson, J. A., Coalson, T. S., Fischl, B., Andersson, J. L., et al. (2013). The minimal preprocessing pipelines for the Human Connectome Project. *NeuroImage* 80, 105–124. doi:10.1016/j.neuroimage.2013.04.127

Jack, C. R., Bernstein, M. A., Borowski, B. J., Gunter, J. L., Fox, N. C., Thompson, P. M., et al. (2010). Update on the MRI Core of the Alzheimer's Disease Neuroimaging Initiative. *Alzheimer's & dementia : the journal of the Alzheimer's Association* 6, 212–220. doi:10.1016/j.jalz.2010.03.004

Marcus, D. S., Fotenos, A. F., Csernansky, J. G., Morris, J. C., and Buckner, R. L. (2010). Open Access Series of Imaging Studies (OASIS): Longitudinal MRI Data in Nondemented and Demented Older Adults. *Journal of cognitive neuroscience* 22, 2677–2684. doi:10.1162/jocn.2009.21407

Nooner, K. B., Colcombe, S. J., Tobe, R. H., Mennes, M., Benedict, M. M., Moreno, A. L., et al. (2012). The NKI-Rockland Sample: A Model for Accelerating the Pace of Discovery Science in Psychiatry. *Frontiers in Neuroscience* 6. doi:10.3389/fnins.2012.00152

Snoek, L., van der Miesen, M. M., Beemsterboer, T., van der Leij, A., Eigenhuis, A., and Steven Scholte, H. (2021). The Amsterdam Open MRI Collection, a set of multimodal MRI datasets for individual difference analyses. *Scientific Data* 8, 85. doi:10.1038/s41597-021-00870-6
